# Adult-Onset Still’s Disease After an mRNA COVID-19 Vaccine in an Older Woman

**DOI:** 10.7759/cureus.51540

**Published:** 2024-01-02

**Authors:** Hiroaki Nishioka, Shogo Shirota

**Affiliations:** 1 Department of General Internal Medicine, Kobe City Medical Center General Hospital, Hyogo, JPN; 2 Department of General internal Medicine, Kobe City Medical Center General Hospital, Hyogo, JPN

**Keywords:** cytokine storm, immune-mediated adverse events, older individual, mrna covid-19 vaccine, adult-onset still’s disease

## Abstract

Adult-onset Still’s disease (AOSD) is an uncommon autoinflammatory disorder without a clear etiology that primarily affects young adults. New-onset disease at > 80 years of age is uncommon. We present the case of an 82-year-old woman with AOSD which developed after receiving a messenger ribonucleic acid (mRNA) coronavirus disease 2019 (COVID-19) vaccine. COVID-19 vaccines are known to cause overproduction of cytokines, systemic inflammation, and some immune-mediated adverse events, such as rheumatoid arthritis, systemic lupus erythematosus, dermatomyositis, vasculitis, and polymyalgia rheumatica after the vaccination has been reported. A handful of cases of AOSD after the vaccination have also been reported and the median age was 40s. However, AOSD related to COVID-19 vaccination can develop even in older individuals.

## Introduction

Coronavirus disease 2019 (COVID-19) has spread worldwide and almost seven million deaths have been reported [1], which has forwarded rapid development of new vaccines for COVID-19. These vaccines mainly have two types of preparation: messenger ribonucleic acid (mRNA)-based vaccines, such as BNT162b2 vaccine (Pfizer/BioNTech) and mRNA-1273 vaccine (Moderna), and adenovirus vector vaccines, such as ChAadOx1 nCoV-19 vaccine (AstraZeneca) and Ad26.COV2.S vaccine (Janssen). COVID-19 vaccines have the ability to induce both cellular and humoral immune responses. There have been case reports of new-onset or relapsed rheumatic diseases following COVID-19 vaccination, including rheumatoid arthritis, sarcoidosis, Bechet’s disease, systemic lupus erythematosus, dermatomyositis, vasculitis, and polymyalgia rheumatica [2,3].

We herein report a case of new-onset adult-onset Still’s disease (AOSD) following COVID-19 vaccination in an older woman.

## Case presentation

**Figure 1:**
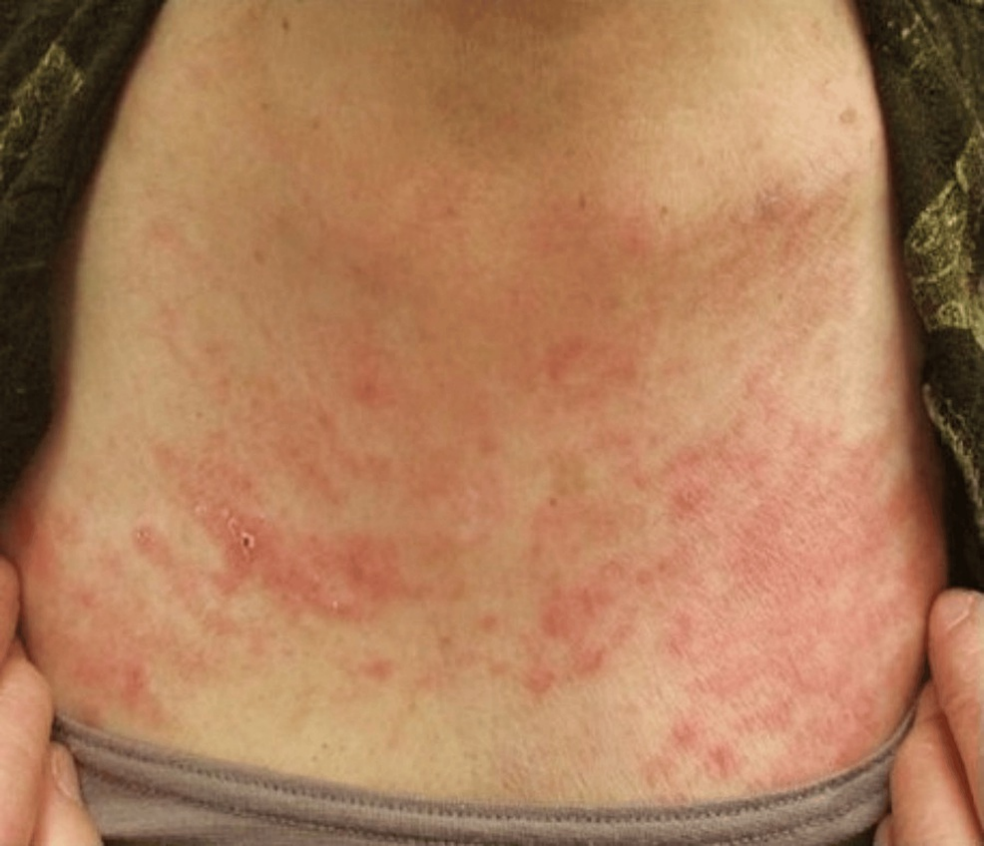
Erythema of the anterior neck.

**Figure 2:**
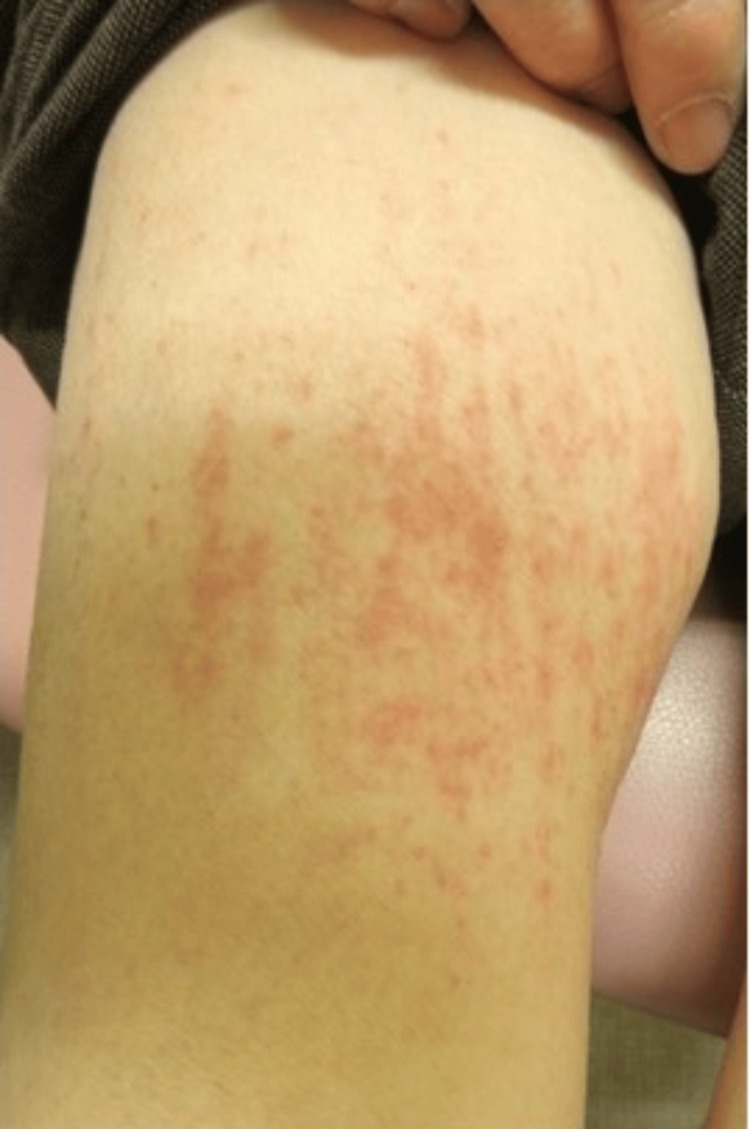
Erythema of the thigh.

An 82-year-old woman was referred to our hospital due to a 20-day history of high-grade fever (39.0 ℃), erythema, and arthralgia at the wrists that developed the day after receiving the fifth dose of mRNA-based vaccine (BNT162b2, Pfizer) for COVID-19. She visited a local clinic and was prescribed antipyretics and antibiotics; however, the symptoms did not improve. Her medical history included hypertension and diabetes mellitus, and she was taking amlodipine and alogliptin/metformin. Physical examination revealed erythema on the anterior neck (Figure 1), chest wall, and thighs (Figure 2). Her wrist joints were swollen, red, and tender, and her fingers were swollen. Laboratory tests showed the following: a white blood cell (WBC) count of 16,800 /μL, neutrophils of 88.5%, a C-reactive protein CRP level of 14.5 mg/dL, and a ferritin level of 66,994 ng/mL (Table 1).

**Table 1:** Laboratory examination.

	Result	Normal range
White blood cell (/μL)	16,800	3,900–9,800
Aspartate transaminase (U/L)	197	8–40
Alanine aminotransferase (U/L)	70	8–40
C-reactive protein (mg/dL)	14.5	0–0.50
Ferritin (ng/mL)	66,994	25-250

Test results for antinuclear antibodies and rheumatoid factor were negative. Blood culture did not show any bacteria. No specific abnormalities were observed on chest or abdominal computed tomography scans. Skin biopsy of the right thigh revealed superficial perivascular dermatitis with neutrophil infiltration; no malignant cells were detected.

Based on the Yamaguchi classification criteria [4], the patient was diagnosed with AOSD. We initiated treatment with intravenous methylprednisolone (1 g/day for three days) and prescribed oral prednisolone 40 mg/day (1 mg/kg/day). Her fever subsided, and erythema diminished. The WBC count and CRP and ferritin levels also decreased. We gradually reduced the prednisolone dose. Recently, the patient was taking prednisolone (6 mg/day), and her symptoms did not recur.

## Discussion

AOSD is an uncommon inflammatory disorder characterized by high fever, redness and swelling of the joints, arthralgia, and a salmon-colored rash on the trunk and extremities. Other features include leukocytosis with neutrophilic predominance, anemia, elevated acute-phase reactant levels, and elevated ferritin concentrations. AOSD mostly affects young adults with the first peak between 15 and 25 years old and the second at 36 to 46 years old, and its new onset at over 80 years is uncommon [5]. 

The new onset of AOSD after COVID-19 vaccination is rare and only 24 patients have been reported [3,6,7]. The median age was 48.5 years. Fourteen cases were related to mRNA-based vaccines and ten cases to viral vector vaccines. The period from vaccination to the onset of AOSD varied from 1 day to 4 months after the first or second dose. All patients improved with the use of immunomodulatory agents. Our patient was much older than in previous cases and developed AOSD after the fifth dose. This may be related to the fact that CD4+ T cell responses are impaired in older adults, which is associated with reduced immunogenicity and reactogenicity of mRNA COVID-19 vaccination [8].

The pathogenesis of AOSD is considered to involve deviant activation of the innate immune system, resulting in a cytokine storm with high concentrations of interleukin (IL)-1, IL-6, and IL-8 [5]. SARS-CoV-2 spike protein in COVID-19 vaccines is thought to induce overproduction of cytokines and systemic inflammation via toll-like-receptor-mediated pathways and inflammasome pathways [9]. Immunological activation by COVID-19 vaccines is presumed to be involved in the triggering of AOSD.

## Conclusions

Although the new onset of AOSD at over 80 years is uncommon, this case demonstrates that AOSD can be induced by COVID-19 vaccination even in older individuals. Nowadays COVID-19 vaccines have been widely used. When patients present with persistent fever and rash, clinicians should consider AOSD as a possible differential diagnosis even in older individuals. It will be necessary to study a number of cases to clarify the risk of COVID-19 vaccines regarding AOSD development. However, AOSD is a rare disease and the use of COVID-19 vaccines should not be avoided in the present COVID-19 pandemic.
